# Managing acute stroke in low-resource settings

**DOI:** 10.2471/BLT.15.162610

**Published:** 2016-06-02

**Authors:** Aaron L Berkowitz

**Affiliations:** a Department of Neurology, Brigham and Women’s Hospital, Harvard Medical School, 75 Francis Street, Boston, MA 02445, United States of America.

Providing appropriate management to patients with acute stroke depends on the underlying etiology of the stroke. Current guidelines from the American Heart Association and American Stroke Association rely on computed tomography (CT) scans to distinguish between acute ischaemic stroke^1^ and acute intracerebral haemorrhage.^2^ Yet the majority of strokes worldwide (around 70% of approximately 17 million per annum) occur in low- and middle-income countries^3^ with limited access to CT. Global data on the availability of medical devices in 2014 estimated the number of CT scanners per 1 million population as only 0.32 in low-income countries compared with 42 in high-income countries.^4^ Moreover, neurodiagnostic tests are often inaccessible or unaffordable to many patients in low-income settings.^5^

Stroke-related disability and mortality are higher in low- and middle-income countries compared with high-income countries ^3^ One potential reason for these poorer outcomes may be uncertainty among physicians about how best to manage patients presenting with acute stroke when CT is unavailable to distinguish ischaemic from haemorrhagic stroke. This paper outlines some considerations in treating patients with acute stroke of unknown etiology in settings where CT is unavailable. These recommendations are based on existing data regarding management of acute ischaemic stroke and acute intracerebral haemorrhage in high-resource settings, epidemiological data, data from decision analyses, and clinical decision rules.

Many aspects of supportive care are the same for acute ischaemic stroke and acute intracerebral haemorrhage, including maintenance of euglycemia and euthermia, provision of adequate hydration and nutrition, treatment of seizures if they occur, prevention of aspiration, prevention of deep-vein thrombosis, and early mobilization of the patient.^1^^,^^2^ Where pneumatic compression is unavailable, prophylaxis of deep-vein thrombosis with low-dose heparin appears to be safe to initiate as early as day 2 after acute intracerebral haemorrhage,^6^ and so could likely be safely initiated at this time in patients with stroke of unknown etiology. Improving these basic aspects of comprehensive stroke care could be achieved through educational initiatives for front-line providers in low-resource settings. In such settings, these basic aspects of supportive care may be more important for stroke outcomes than the two aspects of acute stroke management that differ between haemorrhagic and ischaemic stroke: blood pressure management and use of antithrombotic therapy.

Lowering systolic blood pressure is recommended for patients with acute intracerebral haemorrhage who present with elevated blood pressure;^2^ reduction to below 140 mmHg appears safe but is of uncertain benefit.^7^ After acute ischaemic stroke, blood pressure is often allowed to autoregulate unless thrombolytic therapy is administered.^8^ In cases of acute ischaemic stroke in which intravenous tissue-type plasminogen activator is administered, blood pressure is subsequently maintained below 180/105 mmHg,^1^ and lowering blood pressure in this setting does not appear to negatively affect the outcomes of acute ischaemic stroke.^9^ Therefore, when CT is not available to distinguish between haemorrhagic and ischaemic stroke, it may be reasonable to consider lowering systolic blood pressure to below 180 mmHg for all patients with acute stroke of unknown etiology. This will benefit patients with acute intracerebral haemorrhage and should be safe in patients with acute ischaemic stroke based on studies of the use of intravenous tissue-type plasminogen activator for acute ischaemic stroke.^9^ In the rare cases of patients with stroke of unknown etiology who worsen clinically when blood pressure is lowered, blood pressure could be raised with a bolus of intravenous normal saline and then allowed to autoregulate.

When CT is not available, intravenous tissue-type plasminogen cannot be safely administered, and aspirin is generally the only antithrombotic agent available. The combined results of two large trials – the International Stroke Trial (IST) and the Chinese Acute Stroke Trial (CAST) – demonstrated that daily aspirin (300 mg in IST; 160 mg in CAST) initiated within the first 48 hours after acute ischaemic stroke decreased the risks of recurrent ischaemic stroke and of in-hospital death compared with placebo, despite a small increase in the risk of acute intracerebral haemorrhage.^10^ Patients taking aspirin at the time of acute intracerebral haemorrhage have been shown to have an increased risk of death,^11^ but the risk of initiating aspirin in patients with acute intracerebral haemorrhage has not been formally studied. It is generally presumed that aspirin would be harmful in cases of acute intracerebral haemorrhage. Therefore, many practitioners in settings without access to CT do not administer aspirin to any patients with acute stroke of unknown etiology due to concern that acute intracerebral haemorrhage, if present, could worsen. This may explain in part why only 3.8% of 346 patients studied in four low-income countries were found to be on antiplatelet agents for secondary prevention after ischaemic stroke compared with 53.1% of 213 patients in three high-income countries.^12^

An alternative to the risk-averse strategy of avoiding aspirin in all patients with acute stroke of unknown etiology would be to give aspirin to all of these patients when neuroimaging is unavailable. The risk of this strategy depends in part on what percentage of acute strokes are ischaemic or haemorrhagic. The highest reported proportion of strokes due to intracerebral haemorrhage in a large epidemiological study was 34% (in sub-Saharan Africa), with a range of 9–26% across other world regions.^13^ Although smaller studies have reported the proportional incidence of intracerebral haemorrhage to be as high as 60% in sub-Saharan Africa,^14^^–^^16^ those results must be interpreted cautiously given that the severity of illness may be greater in referral centres with the capacity to perform CT scans and that patients with minor symptoms may not present for evaluation to such centres. Treating all patients with acute stroke with aspirin where CT is unavailable could therefore be beneficial to at least two thirds of the population of stroke patients.

The risk to up to one third of the remaining patients whose strokes are caused by acute intracerebral haemorrhage is difficult to quantify. Across the IST and CAST trials, 773 patients with acute intracerebral haemorrhage (out of the total of 40 000 patients across the two trials) were inadvertently randomized to aspirin or placebo with no difference in outcome, although the trials were not specifically designed to assess this population and the dosage of aspirin administered to this group was not reported.^10^ A decision analysis based on these data suggests that the strategy of administering aspirin to all patients with acute stroke of unknown etiology may hold less risk than perceived, and may even be beneficial regardless of the proportion of acute strokes due to acute intracerebral haemorrhage.^17^

The benefit of aspirin for acute ischaemic stroke in the IST and CAST trials was seen when aspirin was initiated within the first 48 hours after acute ischaemic stroke,^10^ and the highest risk of acute intracerebral haemorrhage expansion is in the first 24 hours.^18^ Therefore, initiating aspirin 25–48 hours after an acute stroke of unknown etiology could minimize the risk to patients with acute intracerebral haemorrhage while preserving the benefit to those with acute ischaemic stroke.^17^ Since aspirin dosages of both 160 mg and 300 mg were found to be effective in IST and CAST, the lower dose of aspirin could be used for the first 2–4 weeks after stroke of unknown etiology, before reducing it to a long-term secondary prevention dose (e.g. 81–100 mg daily).^17^^,^^19^

Clinical decision rules could be used to determine which patients are more likely to have acute ischaemic stroke versus acute intracerebral haemorrhage, although these rules have been found to have limited predictive capacity.^20^ The presence of coma, neck stiffness, seizures, diastolic blood pressure above 110 mmHg, vomiting, and headache are suggestive of acute intracerebral haemorrhage, whereas carotid bruit, a preceding transient ischaemic attack and an alert level of consciousness suggests that acute ischaemic stroke is more likely.^20^ Notably, a clinician’s overall impression based on these factors appears to be nearly as good as any individual factor for predicting whether an acute stroke is due to acute intracerebral haemorrhage or not.^20^ Clinical factors could therefore be used to identify patients who are more likely to have acute intracerebral haemorrhage, so that more aggressive blood pressure management and avoidance of aspirin could be considered in such patients. In clinically ambiguous situations, physicians should note that acute ischaemic stroke is more common than acute intracerebral haemorrhage, as discussed above.

In parallel with increasing attention to primary and secondary prevention of cardiovascular disease in low and middle-income countries,^21^ efforts should be made to establish best practices for acute stroke care in such settings. An expert panel should be convened to formulate consensus guidelines for the management of acute stroke of unknown etiology in settings where there is no rapid access to neuroimaging to determine the underlying etiology of stroke, as these settings account for a substantial proportion of the world’s stroke patients. Educational programmes for front-line health-care providers, focusing on simple supportive interventions, could improve outcomes in settings where advanced diagnostics and treatment of stroke remain limited.
